# Prognostic value of HMGB1 in early breast cancer patients under neoadjuvant chemotherapy

**DOI:** 10.1002/cam4.827

**Published:** 2016-07-25

**Authors:** Ruth Exner, Monika Sachet, Tobias Arnold, Mercedes Zinn‐Zinnenburg, Anna Michlmayr, Peter Dubsky, Rupert Bartsch, Guenther Steger, Michael Gnant, Michael Bergmann, Thomas Bachleitner‐Hofmann, Rudolf Oehler

**Affiliations:** ^1^ Department of Surgery and Comprehensive Cancer CenterMedical University of ViennaViennaA‐1090Austria; ^2^ Department of Internal Medicine I and Comprehensive Cancer CenterMedical University of ViennaViennaA‐1090Austria

**Keywords:** Breast cancer, chemotherapy, high‐mobility group box 1 protein, immunogenic cell death, prognostic marker

## Abstract

The response to neoadjuvant chemotherapy in breast cancer patients is usually assessed by pCR and RCB score. However, the prognostic value of these parameters is still in discussion. We showed recently that an epirubicin/docetaxel therapy is associated with an increase in the cell death marker high‐mobility group box 1 protein (HMGB1) in the circulation. Here, we investigate whether this increase correlates with the long‐term outcome. Thirty‐six early breast cancer patients under neoadjuvant epirubicin/docetaxel combination chemotherapy were included in this study. To determine the immediate effect of this treatment on HMGB1, we collected blood samples before and 24–96 h after the initial dose. This time course was then compared to the 5‐year follow‐up of the patients. HMGB1 levels varied before chemotherapy between 4.1 and 11.3 ng/mL and reacted differently in response to therapy. Some patients showed an increase while others did not show any changes. Therefore, we subdivided the patient collective into two groups: patients with an at least 1.1 ng/mL increase in HMGB1 and patients with smaller changes. The disease‐free survival was longer in the HMGB1 increase group (56.2 months vs. 46.6 months), but this difference did not reach significance. The overall survival (OS) was significantly better in patients with an increase in HMGB1 (log rank *P* = 0.021). These data suggest that an immediate increase in HMGB1 levels correlates with improved outcome in early breast cancer patients receiving neoadjuvant chemotherapy, and may be a valuable complementary biomarker for early estimation of prognosis.

## Introduction

Neoadjuvant chemotherapy (NCT) is a standard in locally advanced high‐risk early breast cancer, in situations where primary breast conservation appears unlikely/impossible 1, and in certain biological subtypes (TN, HER2+) in which high‐pathologic complete response (pCR) rates can be expected 2, 3. Prognostic factors such as intrinsic subtype and proliferation rate, tumor size, and lymph node involvement help to adapt individual therapy strategy. However, biomarkers allowing early evaluation of therapy response and correlate with prognosis are still missing. More accurate monitoring of early response would open the possibility of adapting therapy early to avoid unnecessary burden of nonactive treatments, and eventually achieve improved response and long‐term prognosis. Response to NCT is currently quantified by pCR and residual cancer burden (RCB) score. Additional parameters differentiating better would—particularly if they were available early in the course of treatment—be required to improve the accuracy of prognosis. In this study, we correlate changes in patient blood levels of the cell death marker HMGB1 during early NCT with the overall and disease‐free survival in comparison with pCR.

Chemotherapy‐induced tumor cells death is associated with the release of intracellular molecules into the microenvironment. This can occur either in the form of passive release in case of necrosis or as an active release during apoptosis, for example, in the form of microparticles 4. Studies in animal models showed that some of these molecules can act as damage‐associated molecular pattern (DAMP) 5, 6, 7. DAMPs activate antigen‐presenting cells (APCs) and induce inflammatory reactions boosting thereby the anti‐cancer immune response, a phenomenon which is termed “immunogenic cell death (ICD).” Many routinely employed anti‐cancer treatments, including various chemotherapeutic drugs and radiotherapy, can induce ICD 8. Thus, such treatments have in addition to their direct cytostatic effect against cancer cells also an indirect effect via stimulation of the anti‐cancer immune response. This is especially important when the direct effect is not sufficient to eliminate every single cancer cell. However, the degree of ICD differs between patients and additional treatment might be necessary for its induction. For a final proof of ICD in a specific patient, it is necessary to take tumor biopsies at different time points before and during cytostatic treatment. But this invasive approach is not suited for routine analysis. A recently published consensus paper defines parameters which can be used as a surrogate marker of ICD in clinical studies 9. This includes the detection of cell surface‐exposed calreticulin, extracellular ATP, and release of the high‐mobility group box 1 protein (HMGB1).

HMGB1 is a nonhistone nuclear factor which binds under physiological conditions strongly to DNA and enhances transcription. In case of cell damage, HMGB1 is passively released from the cell and activates APCs by binding to various receptors, including TLR2, TLR4, TLR9, and the receptor for advanced glycosylation products (RAGE) 10, 11, 12. Increased blood levels of HMGB1 have been observed in different disease states, including cancer 13, septic shock 14, rheumatoid arthritis 15, and acute liver injury 16.

In a previous study, we showed that epirubicin/docetaxel combination chemotherapy induces an increase in HMGB1 in the peripheral blood of early breast cancer patients within a few days after administration of chemotherapy 17. The therapy was given in six cycles (every 3 weeks for a total duration of 18 weeks). All patients underwent surgery even if there was no tumor left. In a patient subgroup, we observed an up to twofold increase in HMGB1 blood levels within the first few days of the initial dose of therapy. Importantly, such an increase was only detected in patients who eventually showed pathological tumor regression after the end of the entire treatment. Patients with later pCR showed the strongest early increase in plasma HMGB1 levels after the first dose of therapy. The later time course of HMGB1 was not measured. Thus, the increase in HMGB1 in the peripheral blood during the first few days of chemotherapy can be indicative for the patient's response to chemotherapy. The current study investigates whether chemotherapy‐induced changes in HMGB1 blood levels observed in our previous study correlate with the long‐term outcome. Therefore, we subdivided the patient collective exclusively according to the HMGB1 levels, and compared disease‐free and overall survival of these subgroups.

## Material and Methods

### Patient populations

This study included the same 41 patients with biopsy‐proven breast cancer as in our previous study 17. Patients were enrolled between November 2005 and November 2008. The 5‐year follow‐up data could be retrieved for 36 (88%) patients. For patient characteristics, see Table S1. The therapeutic regimen consisted of six cycles of epirubicin (75 mg/m^2^) and docetaxel (75 mg/m^2^) (every 3 weeks for a total duration of 18 weeks) with the addition of G‐CSF support. Eleven patients also received in addition oral capecitabine (1000 mg/m^2^ for 2 weeks followed by 1 week of rest) as part of the prospective randomized Austrian Breast Cancer Study Group trial 24 18. Four patients also received trastuzumab because of the HER‐2 positivity of their disease. Response to chemotherapy was assessed every three cycles by magnetic resonance imaging using the WHO criteria 19. pCR was determined by histopathology and defined as absence of invasive tumor in primary cancer as well as axillary lymph nodes 20. All patients had appropriate staging before study entry to exclude metastasis by CT and bone scan. The study was approved by the local ethics committee of the Medical University of Vienna and all participants have given informed consent.

### Sample collection and assays

Blood collection and plasma preparation were performed as described previously 3. Blood samples were taken before as well as between day 1 and day 4 after first administration of NCT. The exact day of blood collection had no statistical significant influence on the results 17. Quantification of HMGB1 in the plasma samples was assessed by an enzyme‐linked immunosorbent assay (ELISA) kit (IBL Hamburg, Hamburg, Germany). Lactate dehydrogenase (LDH) levels were quantified using an LDH assay from Biovision (Mountain View, CA). Soluble forms of the checkpoint molecules CD27, CD28, CD80/B7‐1, CD137, CD152/CTLA‐4, CD223/LAG‐3, CD270/HVEM, CD272/BTLA, CD273/PD‐L2, CD274/PD‐L1, CD279/PD‐1, GITR, IDO, and TIM‐3 were measured using a ProcartaPlex^®^ Human Immuno‐Oncology Checkpoint Panel (Affymetrix, Santa Clara, CA).

### Statistics

The sample size calculation for this study was based on the hypothesis that the therapy affects the plasma HMGB1 levels in the group of patients surviving at least 5 years after therapy. For estimation of the biological variation in plasma HMGB1 levels, we used the results from another study with nine untreated colon cancer patients (*σ *= 1.6; median concentration: 6.8 ng/mL). Based on these values, we calculated that a sample size of at least 16 patients per group is needed to achieve a power of 0.8 (Δ = 1.2 ng/mL, *α *= 0.05) 21. We included 36 patients in the study, who were subdivided into two equal subgroups (*n* = 18 and *n* = 18) according to the observed increase in plasma HMGB1. For comparison of HMGB1 levels between the experimental groups, an unpaired Student's *t*‐test was used. To evaluate the increase in HMGB1 in each patient, a paired Student's *t*‐test was used. The calculations of the Kaplan–Meier curves were made using SPSS Software (Version 17.0, SPSS Inc., Chicago, IL). The study was conducted in accordance to the “REporting recommendations for tumor MARKer prognostic studies” (REMARK) 22.

## Results

### The response to therapy did not correlate with the long‐term survival

We recruited 36 patients with confirmed breast cancer who were scheduled for neoadjuvant epirubicin/docetaxel combination chemotherapy. Their characteristics are shown in Table 1 and Table S1. In a total of 20 (55%) of these patients we could observe a response to this treatment at the end of therapy: 5 (14%) of them showed a pathological complete remission (CR) of the tumor defined as ypT0ypN0 or 4 ypTisypN0, 15 (41%) a partial remission (PR: PT1 N0 or N1a). In the 45% of patients, the tumor remained either stable (28%) or even increased in size (progressive disease, *N* = 6, 17%). All patients were subjected to surgery after the end of chemotherapy. Five years later, the majority of patients (78%) were still recurrence free; only eight patients suffered disease recurrence (one of them developed a contralateral recurrence) and six of them died from breast cancer within the follow‐up period of 5 years. Neither pCR alone nor the combination of CR, NCR, and PR (= clinical benefit rate, CBR) showed any correlation with 5‐year disease‐free survival (DFS) or overall survival (OS) as assessed by cross‐tabulation (Pearson's chi‐square ≥ 0.15 in all comparisons). Because of the low patient number this finding should be viewed with caution.

**Table 1:** Patient Characteristics

Characteristics	Number	Percentage (%)
Female/male	36/0	100/0
Age (median)	51 years	
Range	(26–74)	
Chemotherapies		
ED	21	58
EDT	2	6
EDC	11	30
EDCT	2	6
Therapy response		
CR	5	14
NCR	7	19
PR	8	22
SD	10	28
PD	6	17
5‐year OS		
Yes	30	83
No	6	17
5‐year DFS		
Yes	28	78
No	8	22

	Median	Range
HMGB1		
Before the initial dose	5.82 ng/mL	(4.10–11.30)
In the first 4 days of the initial dose	7.11 ng/mL	(4.05–13.61)
Increase	1.08 ng/mL	(−3.48–7.12)
LDH		
LDH before the initial dose	83.0 mU/mL	(50.9–96.7)
LDH in the first 4 days of the initial dose	82.5 mU/mL	(55.0–94.2)
Increase	2.11 mU/mL	(c)

Chemotherapies consisted of different combinations of epirubicin (E), docetaxel (D), capecitabine (C), and trastuzumab (T); Therapy response: complete remission (CR), near complete remission (NCR), partial remission (PR), stable disease (SD), progressive disease (PD); OS, overall survival; DFS, disease‐ free survival; LDH lactate dehydrogenase.

### The initial dose of chemotherapy increased plasma HMGB1 levels in individual patients

**Figure 1:**
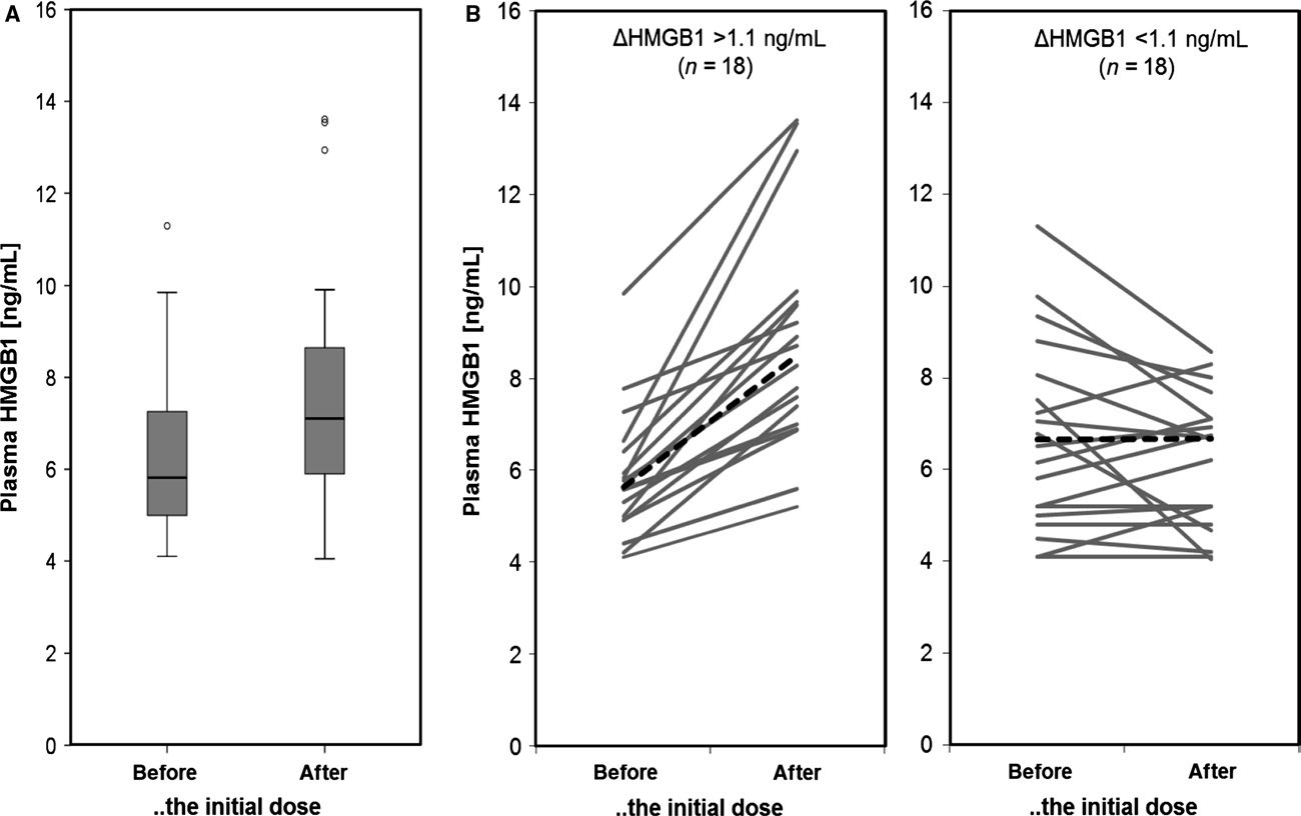
Plasma HMGB1 levels during the initial dose of chemotherapy. HMGB1 plasma levels of breast cancer patients (*n* = 36) treated with epirubicin/docetaxel‐based neoadjuvant chemotherapy. Samples were taken immediately before the initial dose of chemotherapy and between days 1 and 4 after the initial dose. (A) Box plots showing the distribution of HMGB1 levels at the two time points. (B) The graphs indicate the chemotherapy‐induced changes in plasma HMGB1 for every individual patient. The left graph show patients with an increase in plasma HGMB1 of at least 1.1 ng/mL. The right graph shows the other patients.

Plasma levels of HMGB1 before initiation of chemotherapy ranged between 4.1 and 11.3 ng/mL (median 5.82 ng/mL). To monitor a potential release of HMGB1 from dying tumor cells in response to the first chemotherapeutic cycle, we collected an additional blood sample in the days after the initial dose of chemotherapy. HMGB1 levels increased in comparison to the level before the initial dose by up to 7.12 ng/mL (see Fig. 1A). In some patients, however, no increase or even a small decrease was observed.

### Correlation of HMGB1 increase during the initial dose of chemotherapy with prognosis

**Figure 2:**
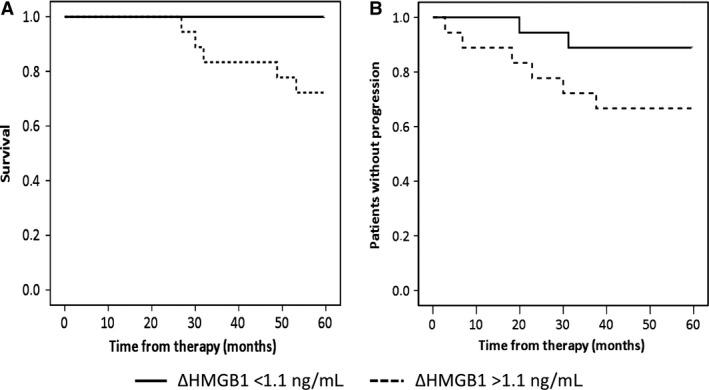
Correlation of HMGB1 with prognosis. Kaplan–Meier curves for overall survival (A) and disease‐free survival (B) based on the increase in HMGB1 in response to the initial dose of therapy (above or below 1.1 ng/mL).

The majority of patients survived 5 years after the neoadjuvant chemotherapy. Based on the plasma HMGB1 levels, we subdivided the patients into two equal subgroups (both *n* = 18): patients with chemotherapy‐induced increase in HMBG1 (ΔHMGB1) of at least 1.1 ng/mL and patients with no or only minimal increase (Fig. 1B). There were no correlations between the two subgroups and other classical tumor covariates (e.g., ER status or TNBC status (as revealed by Pearson's chi‐square test). Figure 2 shows Kaplan–Meier curves of the two HMBG1‐defined study groups for DFS and OS. All patients with a ΔHMGB1 of at least 1.1 ng/mL were still alive 5 years after treatment (Fig. 2A). The group of patients with a lower ΔHMGB1 showed a significantly lower OS (log rank *P* = 0.021) and included all nonsurvivors. The subdivision according to ΔHMGB1 showed a similar trend with regard to DFS which, however, did not reach statistical significance (56.2 months vs. 46.6 months for patients with ΔHMGB1 > 1.1 ng/mL or ΔHMGB1 < 1.1 ng/mL, respectively; log rank *P* = 0.101), which appears to be a numerical issue only because of the limited number of observed events.

### Comparison of HMGB1 with LDH and with the immune status

Next, we determined the plasma levels of LDH, which is an accepted marker for cell death. The analysis revealed that the median LDH levels did not change in response to the initial phase of chemotherapy (see Table 1). However, it changed considerably in individual patients (from −33.1 to 23.2 mU/mL), but without any correlation with HMGB1 (*r* = 0.148). This suggests that the source of HMGB1 might differ from the source of LDH.

**Figure 3:**
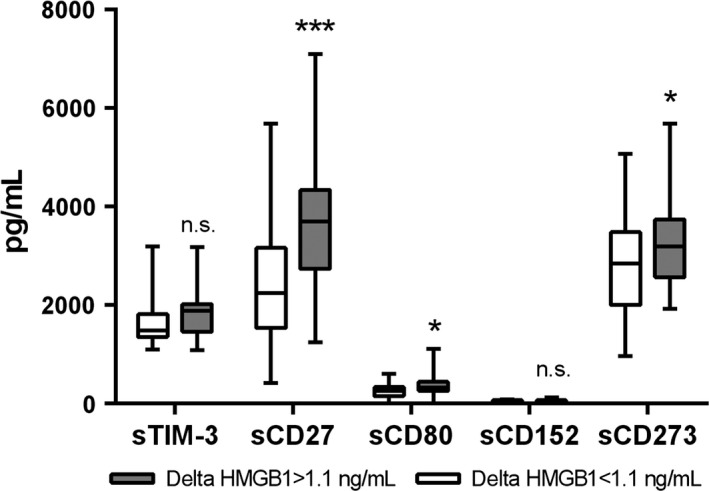
Plasma concentrations of soluble immune checkpoint molecules. Blood was taken from our patient collectively immediately before the initial dose of chemotherapy. The plasma levels of soluble form of the checkpoint molecules CD27, CD28, CD80/B7‐1, CD137, CD152/CTLA‐4, CD223/LAG‐3, CD270/HVEM, CD272/BTLA, CD273/PD‐L2, CD274/PD‐L1, CD279/PD‐1, GITR, IDO, and TIM‐3 were measured using a multiplex immunoassay. The graph indicates the concentration of those molecules that showed quantifiable levels of the respective protein subdivided according to the delta HMGB1 shown in Figure 1. All other proteins were close to or below the detection limit. Statistical significant differences between these two groups were calculated using a Student's *t*‐test are indicated by asterisks (*P < 0.05; *^**^ *P* < 0.002). PD, progressive disease.

In beside of the passive release of HMGB1 during cell death, it can be also actively released from stimulated immune cells such as macrophages. Thus, variations in the immune system activation status of the patients might contribute to the above‐described differences in HMGB1 plasma levels. A well‐known determinant of the responsiveness of the immune system against cancer cells are immune checkpoint molecules. Therefore, we investigated the baseline plasma levels of various soluble checkpoint molecules in the two HMGB1 subgroups. Six immune checkpoint molecules could be detected in the plasma samples of almost all patients (CD27, CD28, CD80/B7‐1, CD152/CTLA‐4, CD273/PD‐L2, and TIM‐3; see Fig. 3 and Table S2). Seven checkpoint molecules were not detectable in most samples (CD137, CD223/LAG‐3, CD270/HVEM, CD272/BTLA, CD274/PD‐L1, CD279/PD‐1, and IDO). Interestingly, CD27, CD80/B7‐1, and CD273/PD‐L2 were higher in the delta HMGB1 ≥ 1.1 ng/mL group (see Fig. 3). This confirms that the immune status differs between patients showing an increase in HMGB1 in response to NCT in comparison to patients without increase. It has to be noted that none of the soluble checkpoint molecules correlated with the response to therapy, OS, or DSF.

## Discussion

In this study, we show that an epirubicin/docetaxel‐induced increase in the blood levels of HMGB1 during the first days of neoadjuvant chemotherapy of early breast cancer correlates with the long‐term survival of patients. This indicates that the earliest response to the very first cytotoxic application can be a valuable tool for prognosis assessment.

The median prechemotherapy plasma HMGB1 level of patients in this study was 5.82 ± 1.8 ng/mL. This is in a similar range as observed by others in patients with mesothelioma (6.7 ng/mL) 23 and in patients with colorectal cancer‐derived liver metastases (7.1 ng/mL) 24. In contrast, the research group of Holdenrieder reported somewhat lower HMGB1 levels in different populations of cancer patients: benign breast cancer (1.7 ng/mL), advanced pancreatic cancer (2.0 ng/mL), advanced stage of hepatocellular carcinoma (1.7 ng/mL), and advanced stage of hepatocellular carcinoma (1.7 ng/mL) 25, 26, 27. The main difference to our study is that we used plasma samples instead of serum samples. In addition, the ELISA method for HMGB1 quantification is not a validated assay for routine analysis. Therefore, we assume that such small differences in the absolute levels between studies are related to technical variations and are not biologically significant.

The HMGB1 levels detected before the initiation of chemotherapy varied between patients (with a CV of 31%). This might be associated to a different degree of necrosis in the primary tumor. The area in the middle of tumor nodes shows frequently an insufficient supply with oxygen and nutrients leading to local necrosis of tumor cells and, thus, release of HMGB1 28. Accordingly, a study on acetaminophen hepatotoxicity showed that high plasma HMGB1 is a predictive indicator of acetaminophen‐induced liver necrosis 29. Similarly, pretherapeutic values of HMGB1 were slightly higher in advanced pancreatic cancer patients when compared to the healthy controls 26. High pretherapeutic values of serum HMGB1 in patients with colorectal cancer liver metastases were associated with poor 2‐year overall survival 24. Similar relationship of high serum HMGB1 and poor OS was observed in patients with mesothelioma 23. These data strongly suggest that high HMGB1 correlate with the tumor load and with tissue damage. But pretreatment levels have not to be confused with treatment‐induced changes in HMGB1 levels. We observed an increase in HMGB1 within the first days of the initial chemotherapy cycle in patients with a better prognosis. A modified but similar kinetic of plasma HMGB1 levels was observed by others in breast cancer patients with locally confined breast cancer receiving neoadjuvant epirubicin/cyclophosphamide followed by docetaxel chemotherapy 25. Patients without any response to treatment showed a significant decrease in HMGB1 within the first cycle of therapy. This decrease was much less pronounced in patients with either partial remission or complete remission. Unfortunately, no data on the long‐term survival are available from that study. In difference to our study, the authors determined the HMGB1 levels before the start of the second cycle of therapy, that is, 21 days after start of the first cycle and not during the first days. This might be the reason for the observed lower HMGB1 levels. Fahmueller and coworkers showed in patients with liver metastases of colorectal cancer that serum HMGB1 increases as early as within 24 h in response to radioembolization 24. This increase was significantly higher in patients with progressive disease than in nonresponding patients (as assessed 3 months later). Similarly, high HMGB1 levels at days 21 and 56 in patients with advanced pancreatic cancer undergoing chemotherapy were confirmed to indicate short OS 26. Taken together, these studies confirm that chemotherapeutic treatment of cancer patients leads to changes in the blood levels of HMGB1. They suggest the actually observed level of HMGB1 depends strongly on the time point when the sample was collected. The strongest effect of the treatment seems to be visible within the first few days of drug administration. In accordance to our results, these studies show that early dynamics of HMGB1 levels during chemotherapy can be of prognostic value. However, the correlation appears to differ between the various cancer types. Most of these studies were conducted in metastatic patients with a large tumor load in contrast to our study conducted in early stage breast cancer patients. Eventually, more studies including various time points for sample collection and higher sample sizes are needed to get a broader picture of the role of HMGB1.

Circulating HMGB1 is generally regarded as a DAMP which is passively released from dying cells. Thus, the chemotherapy‐induced increase in plasma HMGB1 observed in surviving patients might be related to a higher degree of cell death in response to therapy than in nonsurviving patients. However, plasma LDH, which is a well‐accepted marker of cell death, correlated neither with HMGB1 nor with the long‐term outcome. Thus, plasma HMGB1 might not exclusively derive from dying tumor cells. HMGB1 was shown to be also released actively from stimulated immune cells such as macrophages 28. Indeed, patients with chemotherapy‐induced increase in HMGB1 showed already before therapy higher levels of soluble forms of the immune checkpoint molecules CD27, CD80/B7‐1, and CD273/PD‐L2 indicating a difference in their immune status. CD27 showed the largest difference. It is a costimulatory receptor expressed on T‐cells and subsets of B and NK cells which interacts with CD70 on APC 30. It can be released as a soluble 32‐kDa form (sCD27) into the circulation most likely generated by proteolytic cleavage from the T‐cell surface after stimulation 30, 31. Correspondingly, increased serum levels of sCD27 have been reported in different malignancies including acute lymphoblastic leukemia, chronic lymphocytic leukemia, and malignant lymphoma 31. Recent studies showed that sCD27 is associated with the response to chemotherapy in B‐cell lymphoma and non‐Hodgkin lymphoma 32. CD80/B7‐1 is found on monocytes and activated B‐cells and interacts with CD28 or CD152/CTLA‐4 on T‐cells. The soluble form of CD80/B7‐1 (sCD80) derives from an alternative splicing variant 33. High levels of sCD80 restore T‐cell activation and help to overcome tumor PDL1‐mediated immune suppression 34. The inhibitory CD273/PD‐L2 is expressed on alternatively activated (M2‐like) macrophages and activated CD4^+^ and CD8^+^ T‐cell subsets. It is able to downregulate cytokine production and proliferation of T‐cells 35. Soluble CD273/PD‐L2 splice variants have been described, but their biological function is still unknown 36. Thus, high levels of sCD27 and sCD80 observed in the present study in some breast cancer patients before therapy indicate an activated and unbreaked immune system. These patients showed a strong increase in HMGB1 in response to chemotherapy and a better prognosis. Patients with no HMGB1 increase showed lower pretreatment values for sCD27, sCD80, and sCD273. These data suggest that the immune system contributes to the HMGB1 found in our patient cohort.

HMGB1 which is actively released from immune cells differs from that passively released from dying tumor cells in regard of its oxidation status: the passively released HMGB1 is fully oxidized, while the actively released form has several accessible free thiol groups on its surface. Unfortunately, the ELISA used here did not differentiate between these two forms. Thus, the question of a causative relationship between the stimulated immune system and the chemotherapy‐induced HMGB1 release remains to be investigated.

Pathological complete response has been proposed as a surrogate endpoint for prediction of long‐term clinical benefit 37. In our study with its limited sample size and a rather heterogenous group of patients, we were unable to show a clear correlation of chemotherapy response quantified by pCR and overall survival. In a recently published study by Cortazar et al., the authors showed that the association between pCR and long‐term outcome was stronger in patients with high‐grade tumors (triple‐negative) than in those with low‐grade tumors 38. About 60% of patients included in the current study showed ER+ tumors, which may result in a relatively poor pCR/outcome correlation. Additional corroboration for our results comes from a recent large metaregression analysis of 29 heterogeneous neoadjuvant trials including 14,641 patients 39. It investigated the role of pCR as a surrogate endpoint for DFS and OS in patients with breast cancer and showed only a weak relationship between the treatment effect on the pCR and the treatment effect on the clinical outcomes. Thus, there is still a need for complementary prognostic biomarkers such as the immediate response of HMGB1 to chemotherapy.

In summary, with the limitation of a relatively small sample size in this pivotal study, our data suggest that an early increase in HMGB1 levels in response to the first cycle of neoadjuvant chemotherapy may correlate with improved long‐term outcome in early stage breast cancer patients receiving neoadjuvant chemotherapy. The fact that LDH did not predict pCR, and subsequently did not predict outcome and also the overall did not predict outcome, then it is almost logical that HMGB1 can be regarded as specific biomarker. If confirmed, this finding has huge potential implications for treatment refining and optimization. We therefore suggest that the prognostic role of HMGB1 should be investigated in future studies.

## Conflict of Interest

The authors declare that they have no competing interests.

## Supporting information


**Table S1.** Individual tumor parameters and HMGB1 levels.

**Table S2.** Individual levels of soluble immune check point molecules.
