# Supplementary material for: Prognostic value of HMGB1 in early breast cancer patients under neoadjuvant chemotherapy
Source: Cancer Med. 2016 Jul 25;5(9):2350–8. doi: 10.1002/cam4.827 (PMC5055166; doi:10.1002/cam4.827)
Supplement: Supplementary file 1 — Table S1. Individual tumor parameters and HMGB1 levels. [file CAM4-5-2350-s001.pdf]

Table S1: Individual tumor parameters and HMGB1 levels

Pat.ID	pT	pN	ER	PR	HER-2	Grading	Therapy	Response	HMGB-1 (ng/ml)		LDH (mU/ml)	
									before	after	before	after
4	4	2a (9/15)	+	+	-	2	ED	SD	5,20	6,20	72,4	77,6
8	is	0 (0/18)	+	-	-	3	ED	CR	4,90	7,80	79,7	83,9
10	NA	NA	+	-	-	2	ED	NCR	4,20	7,40	82,4	94,2
11	1a	0 (0/17)	-	-	-	3	EDC	NCR	6,40	9,91	82,6	84,6
12	2	1a (1/21)	+	+	-	2	ED	SD	4,10	5,20	85,6	88,3
13	1c	1a (1/19)	-	-	amplified	3	EDCT	PR	5,82	12,94	66,0	85,9
14	1c	0 (0/25)	-	-	-	3	ED	PR	5,60	7,00	84,6	81,3
23	1a	1a (2/18)	+	-	-	3	EDC	NCR	5,94	9,67	76,9	75,2
24	3	0 (0/16)	-	-	-	3	EDC	PD	6,78	4,67	84,1	91,5
29	2	1a (1/10)	-	-	-	3	ED	SD	5,30	7,60	78,6	89,7
30	1b	1a (2/11)	-	-	-	3	EDC	PR	6,64	13,54	88,3	86,3
31	1c	2a (9/14)	+	-	-	2	ED	SD	5,20	5,20	71,7	74,9
32	is	0 (0/17)	+	-	amplified	3	EDC	CR	7,27	8,72	91,5	93,7
33	2	0 (0/20)	-	-	-	3	ED	SD	5,00	9,60	85,5	83,0
35	0	0	-	-	-	3	ED	CR	5,66	8,91	79,3	82,9
36	3	0/12	+	+	-	3	EDC	PD	7,24	8,30	85,6	81,0
37	is	0 (0/14)	-	-	-	3	EDC	CR	6,16	7,11	87,0	89,7
38	1a	0 (0/24)	+	+	amplified	3	EDCT	NCR	9,85	13,61	83,6	79,3
39	0	1a (1/17)	-	-	-	3	EDC	NCR	8,81	8,01	79,7	81,9
40	1c	1a (2/21)	-	-	-	3	ED	PR	4,80	4,80	74,9	60,0
47	1b	0 (0/10)	+	+	-	2	ED	NCR	6,52	6,92	84,2	76,7
48	3	2a (8/15)	+	-	-	1	ED	PD	4,50	4,20	96,7	93,5
50	2	1a (2/10)	-	-	-	3	ED	SD	4,99	5,19	80,2	90,8
51	2	0 (0/24)	+	-	-	1	EDC	SD	9,34	7,68	86,6	55,6
58	is	0 (0/16)	+	+	amplified	2	EDT	CR	5,58	6,89	83,7	77,8
60	1c	0	-	-	-	3	ED	SD	8,06	6,66	83,4	85,6
62	1c	0 (0/14)	+	+	-	2	EDC	PR	9,78	7,10	87,0	62,9
63	1b	1a (1/31)	+	+	-	1	EDC	PR	5,77	8,28	60,2	83,4
66	1a	0	-	-	-	3	ED	NCR	4,92	6,88	78,3	78,9
70	1c	2a (4/9)	+	-	amplified	2	EDT	PR	4,10	4,10	83,4	75,0
71	2	1a (1/10)	+	-	-	2	ED	SD	4,40	5,60	73,7	87,1
72	2	1a (1/12)	+	+	-	2	ED	PD	7,78	9,21	80,7	82,1
73	2	0	+	+	-	3	ED	SD	11,30	8,57	86,2	71,4
75	1c	0 (0/5sn)	+	-	-	1	ED	PR	7,53	4,05	50,9	55,0
77	3	1a (2/10)	+	+	-	2	ED	PD	5,81	6,75	72,5	82,0
80	3	3a (12/15)	-	-	-	2	ED	PD	7,06	6,70	84,2	84,5
