# Supplementary material for: Prognostic value of HMGB1 in early breast cancer patients under neoadjuvant chemotherapy
Source: Cancer Med. 2016 Jul 25;5(9):2350–8. doi: 10.1002/cam4.827 (PMC5055166; doi:10.1002/cam4.827)
Supplement: Supplementary file 2 — Table S2. Individual levels of soluble immune check point molecules. [file CAM4-5-2350-s002.pdf]

Table S2: Individual levels of soluble immune check point molecules

Pat.ID	CD27 (pg/ml)	CD28 (pg/ml)	CD80 B7-1 (pg/ml)	CD137 (pg/ml)	CD152 CTLA-4 (pg/ml)	CD223 LAG-3 (pg/ml)	CD270 HVEM (pg/ml)	CD272 BTLA (pg/ml)	CD273 PD-L2 (pg/ml)	CD274 PD-L1 (pg/ml)	CD279 PD-1 (pg/ml)	GITR (pg/ml)	IDO (pg/ml)	TIM-3 (pg/ml)
4	1909,2	n.d.	548,4	n.d.	n.d.	n.d.	n.d.	n.d.	5071,8	n.d.	n.d.	n.d.	n.d.	3196,4
8	2567,3	258,4	300,1	n.d.	53,1	n.d.	n.d.	n.d.	3722,3	n.d.	n.d.	n.d.	n.d.	1381,7
10	3780,2	684,2	279,3	n.d.	127,8	99,4	134,9	n.d.	2263,1	n.d.	n.d.	n.d.	n.d.	2005,9
11	4100,1	200,7	268,9	n.d.	41,2	n.d.	n.d.	n.d.	3704,1	n.d.	n.d.	n.d.	n.d.	2047,2
12	3940,2	369,2	424,1	n.d.	73,2	n.d.	n.d.	n.d.	2659,7	n.d.	n.d.	n.d.	n.d.	1890,2
13	4539,2	258,4	492,3	n.d.	58,6	n.d.	n.d.	n.d.	5683,4	n.d.	n.d.	n.d.	n.d.	2203,9
14	1249,9	n.d.	254,9	n.d.	n.d.	n.d.	n.d.	n.d.	2848,6	n.d.	n.d.	n.d.	n.d.	1560,6
23	7093,7	314,0	1113,8	n.d.	53,1	n.d.	90,5	3865,0	3056,1	n.d.	n.d.	n.d.	n.d.	3174,4
24	2561,6	n.d.	265,4	n.d.	41,2	n.d.	n.d.	n.d.	2907,1	n.d.	n.d.	n.d.	n.d.	1810,9
29	n.m.	n.m.	n.m.	n.m.	n.m.	n.m.	n.m.	n.m.	n.m.	n.m.	n.m.	n.m.	n.m.	n.m.
30	3404,1	286,5	626,8	n.d.	77,9	54,0	n.d.	n.d.	3770,0	n.d.	n.d.	n.d.	n.d.	2380,0
31	2278,1	229,5	265,3	n.d.	61,2	n.d.	n.d.	n.d.	3480,0	n.d.	n.d.	n.d.	n.d.	1832,0
32	4974,7	342,1	361,7	n.d.	63,6	n.d.	n.d.	n.d.	3614,5	n.d.	n.d.	n.d.	n.d.	1973,9
33	2599,5	258,4	368,4	n.d.	53,1	n.d.	n.d.	n.d.	3189,4	n.d.	n.d.	n.d.	n.d.	1982,1
35	2641,3	n.d.	455,7	n.d.	73,2	n.d.	n.d.	n.d.	4778,1	n.d.	n.d.	n.d.	n.d.	1671,8
36	4160,4	258,4	452,5	n.d.	41,2	n.d.	n.d.	n.d.	2444,6	n.d.	n.d.	n.d.	n.d.	1859,9
37	1967,3	n.d.	233,8	n.d.	41,2	n.d.	n.d.	n.d.	3618,5	n.d.	n.d.	n.d.	n.d.	1560,9
38	3228,5	n.d.	368,4	n.d.	n.d.	n.d.	n.d.	n.d.	2823,2	n.d.	n.d.	n.d.	n.d.	1085,7
39	3304,6	423,3	605,6	n.d.	86,5	n.d.	130,6	n.d.	2176,4	n.d.	n.d.	n.d.	91,6	1838,4
40	1395,8	n.d.	n.d.	n.d.	41,2	n.d.	n.d.	n.d.	1566,8	n.d.	n.d.	n.d.	n.d.	1338,5
47	2659,2	200,7	n.d.	n.d.	41,2	n.d.	n.d.	n.d.	959,7	n.d.	n.d.	n.d.	n.d.	1234,8
48	588,2	229,5	341,2	n.d.	53,1	n.d.	n.d.	n.d.	3513,4	n.d.	n.d.	n.d.	n.d.	1712,9
50	3115,2	328,2	298,4	n.d.	77,6	n.d.	81,3	n.d.	3380,5	n.d.	n.d.	n.d.	n.d.	1465,8
51	2498,6	300,6	n.d.	n.d.	63,6	n.d.	n.d.	n.d.	2652,0	n.d.	n.d.	n.d.	n.d.	1550,9
58	5159,8	383,2	324,1	n.d.	63,6	51,7	95,0	n.d.	2477,5	n.d.	n.d.	n.d.	n.d.	1498,3
60	1580,2	229,5	336,1	n.d.	53,1	n.d.	n.d.	n.d.	2961,8	n.d.	n.d.	n.d.	n.d.	1173,3
62	1598,3	396,7	n.d.	n.d.	77,9	61,3	n.d.	n.d.	1612,7	n.d.	n.d.	n.d.	n.d.	1101,9
63	4141,1	448,1	233,9	n.d.	68,2	n.d.	92,6	n.d.	4234,6	n.d.	n.d.	n.d.	n.d.	1901,9
66	3705,3	314,0	324,1	n.d.	86,5	n.d.	n.d.	n.d.	3630,5	n.d.	n.d.	n.d.	n.d.	1716,1
70	414,4	n.d.	289,7	n.d.	n.d.	n.d.	n.d.	n.d.	2134,1	n.d.	n.d.	n.d.	n.d.	1416,9
71	2829,9	300,3	221,5	n.d.	60,9	n.d.	n.d.	n.d.	1922,8	n.d.	n.d.	n.d.	n.d.	1196,7
72	3459,6	314,6	n.d.	n.d.	53,1	n.d.	n.d.	n.d.	2203,3	n.d.	n.d.	n.d.	n.d.	1431,0
73	5680,2	342,1	205,5	n.d.	71,0	n.d.	90,5	n.d.	2780,3	n.d.	n.d.	n.d.	n.d.	1517,2
75	591,5	n.d.	270,6	n.d.	n.d.	n.d.	n.d.	n.d.	2971,6	n.d.	n.d.	n.d.	n.d.	1367,1
77	2216,6	286,5	230,3	n.d.	58,6	n.d.	n.d.	n.d.	3488,0	n.d.	n.d.	n.d.	n.d.	1396,0
80	3430,3	286,5	212,7	n.d.	68,2	n.d.	88,2	n.d.	1486,6	n.d.	n.d.	n.d.	n.d.	1465,8

n.d. = not detectable; n.m. = not measured
